# Use and reporting of systematic review methodology in EFSA scientific opinions on animal health and welfare

**DOI:** 10.3389/fvets.2025.1594235

**Published:** 2026-01-19

**Authors:** Johann Liesner, Benjamin V. Ineichen, Marianna Rosso

**Affiliations:** 1 Center for Reproducible Science and Research Synthesis, University of Zurich, Zurich, Switzerland; 2 Department of Clinical Research, University of Bern, Bern, Switzerland

**Keywords:** 3Rs principle, evidence synthesis, food safety authority, preclinical research, regulatory science, reporting quality, research rigor

## Abstract

**Introduction:**

Despite the growing use of systematic reviews of animal studies, it remains unclear how often systematic review methodology – such as detailed search strategies, critical appraisal, and protocol registration – is applied in regulatory contexts. We aimed to assess the use and reporting quality of systematic reviews in the European Food Safety Authority’s (EFSA) Scientific Opinions on animal health and welfare.

**Methods:**

We conducted an exploratory study comprising of a retrospective analysis of 151 EFSA Scientific Opinions. We classified the types of studies underpinning these reports, including systematic reviews, literature reviews, and other study designs. Additionally, we assessed the reporting rigor of key systematic review elements.

**Results:**

Literature reviews were the most common study type, present in 126 reports (83%), with 40 reports (27%) applying systematic review methods such as searching multiple databases and reporting clear research questions, inclusion criteria, and study counts. Eleven studies (27%) were explicitly labelled as systematic reviews, with their use increasing over time. Reporting quality varied: 64% listed more than one reviewer, 45% reported a risk of bias assessment, and 36% registered a study protocol.

**Discussion:**

Systematic review methodology is increasingly applied in EFSA’s Scientific Opinions on animal health and welfare. However, methodological rigor and reporting standards remain inconsistent, underscoring the need for improvement to strengthen the reliability and transparency of EFSA’s evidence base.

## Introduction

A systematic review synthesizes existing evidence to address a focused question using explicit, reproducible methods (1). Unlike traditional ‘expert’ reviews, which often lack clear selection and evaluation criteria, systematic reviews follow a strict methodology to minimize bias and maintain scientific integrity (2). This approach includes clearly defining a research question, conducting a comprehensive search for relevant studies, applying transparent inclusion and exclusion criteria for studies, and critically appraising the included studies through a risk of bias assessment. As a result, systematic reviews have gained momentum in the field of animal research (3–5).

Systematic reviews have been key in identifying critical issues in animal research, informing best practice guidelines, reducing research waste, enhancing reproducibility, and informing translational research (6–8). For example, a systematic review comparing treatment effects in animal experiments and clinical trials found that treatments tested in animal models closely mimicking clinical scenarios, such as older animals with comorbidities for stroke drugs, tended to show efficacy in human trials (9). Conversely, treatments tested under artificial scenarios in animals often failed to show efficacy in humans (9). Another example is from pancreatitis research: a human clinical trial found no difference in outcomes between probiotic treatment and controls, with higher mortality in the probiotic group (10). An animal systematic review showed that none of the animal studies used the same probiotics as the clinical trial, and probiotics were often administered before inducing pancreatitis (11).

In addition to the above mentioned benefits, animal systematic reviews are considered animal-free innovations because they maximize the value of existing animal studies and generate new insights without new animal experimentation (12), thereby advancing the 3R principles (Replacement, Reduction, and Refinement) (13). Despite this recognized value, little is known about how systematic reviews are incorporated into regulatory processes.

Among relevant institutions, the European Food Safety Authority (EFSA) plays a central role, as it issues Scientific Opinions that provide risk assessments, responses to emerging issues, and methodological guidance in food and feed safety, animal health, and welfare (14, 15). According to the Regulation (EC) No 178/2002 of the European Parliament and of the Council, scientific opinions “serve as the scientific basis for the drafting and adoption of Community measures in the fields falling within its mission” and were thus chosen as suitable for the present study (16). We focused on EFSA because it constitutes uniquely standardized, transparent, and publicly accessible body of policy-relevant evidence, making it well suited for assessing how systematic review methodology is used in regulatory science. Some of these Scientific Opinions draw on systematic reviews, but the extent and quality of their use have not been systematically assessed. Thus, the objective of this study was to evaluate the reporting quality and rigor of systematic reviews used in EFSA’s Scientific Opinions on animal health and welfare. By mapping and appraising these reviews, we aim to provide evidence on how systematic reviews are applied within EFSA’s work and identify areas where methodological rigor can be strengthened.

## Methods

### Study protocol pre-registration

Prior to conducting our study, we drafted and registered a study protocol on the Open Science Framework (OSF)^1^ (17).

### Study design and research question

The present exploratory study comprised of a retrospective literature review of EFSA scientific opinions with the primary research question: how commonly are these scientific opinions informed by systematic review methodology, and what is the reporting quality of these systematic reviews?

### References fetching and article allocation

A list of articles was manually compiled from the EFSA (15) under the article type “Scientific Opinion” and the categories “Animal Welfare” or “Animal Health.” The categories cannot be identified reliably through PubMed and a PubMed search of “EFSA Journal AND (Animal AND (“Welfare” OR “Health”))” did not identify all relevant publications found on the EFSA website. Therefore, references and full texts were retrieved manually from the EFSA webpage by MR and JL.^2^ We used a manual screening approach, applying predefined inclusion and exclusion criteria. Scientific Opinions were included if they addressed topics within the EFSA “Animal Health” or “Animal Welfare” categories and contained a qualitative or quantitative synthesis of evidence. Publications outside these categories, such as Technical Reports, Guidance Documents, or opinions unrelated to animal health or welfare, were excluded.

All articles were allocated randomly using a random number generator in R. The articles were distributed across two reviewers (MR, JL), with 20% of the articles allocated to both reviewers to assess inter-rater reliability (IRR).

### Data extraction

Reviewers independently extracted data from all the articles fetched as outlined in the data extraction table including animal species. Reviewers were blinded to the studies in which IRR were checked. IRR was assessed at the beginning and during the data collection phase. Any discrepancies were resolved through discussion and, if necessary, with the intervention of a third reviewer (BVI).

One reviewer extracted the data, including the publication year and the types of studies that informed the policy paper:

- Literature review: Narrative or descriptive synthesis without predefined search or selection criteria.
- Systematic review: Explicitly structured review with defined research question, inclusion/exclusion criteria, search across ≥2 databases, and a study flow chart.
- Primary study: Original experimental or interventional research generating new data.
- Observational study: Empirical study observing animals without intervention (e.g., cohort, case–control).
- Database study: Analysis of existing datasets or registries.
- Survey: Structured data collection from respondents via questionnaire or interview.
- Other: Study types not fitting the above categories (e.g., expert elicitation or modelling).

We categorized literature reviews as ‘extensive’ when the authors explicitly described them as such. Systematic reviews were identified either by the authors’ label or by meeting specific criteria: a clear research question, defined inclusion and exclusion criteria, a search across at least two databases, and a study flow chart. We provide an analysis only for the explicitly labelled systematic reviews.

In addition, we extracted methodological items of systematic reviews: whether an a-priori protocol was registered, the number of literature databases searched (if applicable), whether a search string was reported, if more than two reviewers were involved, whether a flowchart was provided, and whether a risk of bias assessment or a meta-analysis was conducted.

### Data analysis and synthesis

We provide a narrative synthesis and descriptive statistics for the extracted data. We used R 4.4.1 for all visualizations (18). All code and data supporting our conclusions are publicly available at the Open Science Framework (see Footnote 1).

### Deviations from the protocol

During the extraction, we observed many studies following the “Animal Health Law” (AHL) framework (19), which we did not anticipate during the conceptualization of this study. This framework provides a specified flowchart of study types for the research to follow, including but not limited to: data collection from scientists to compile fact sheets, expert judgements, and EFSA panels evaluations.

## Results

### Included studies and their characteristics

**Figure 1:**
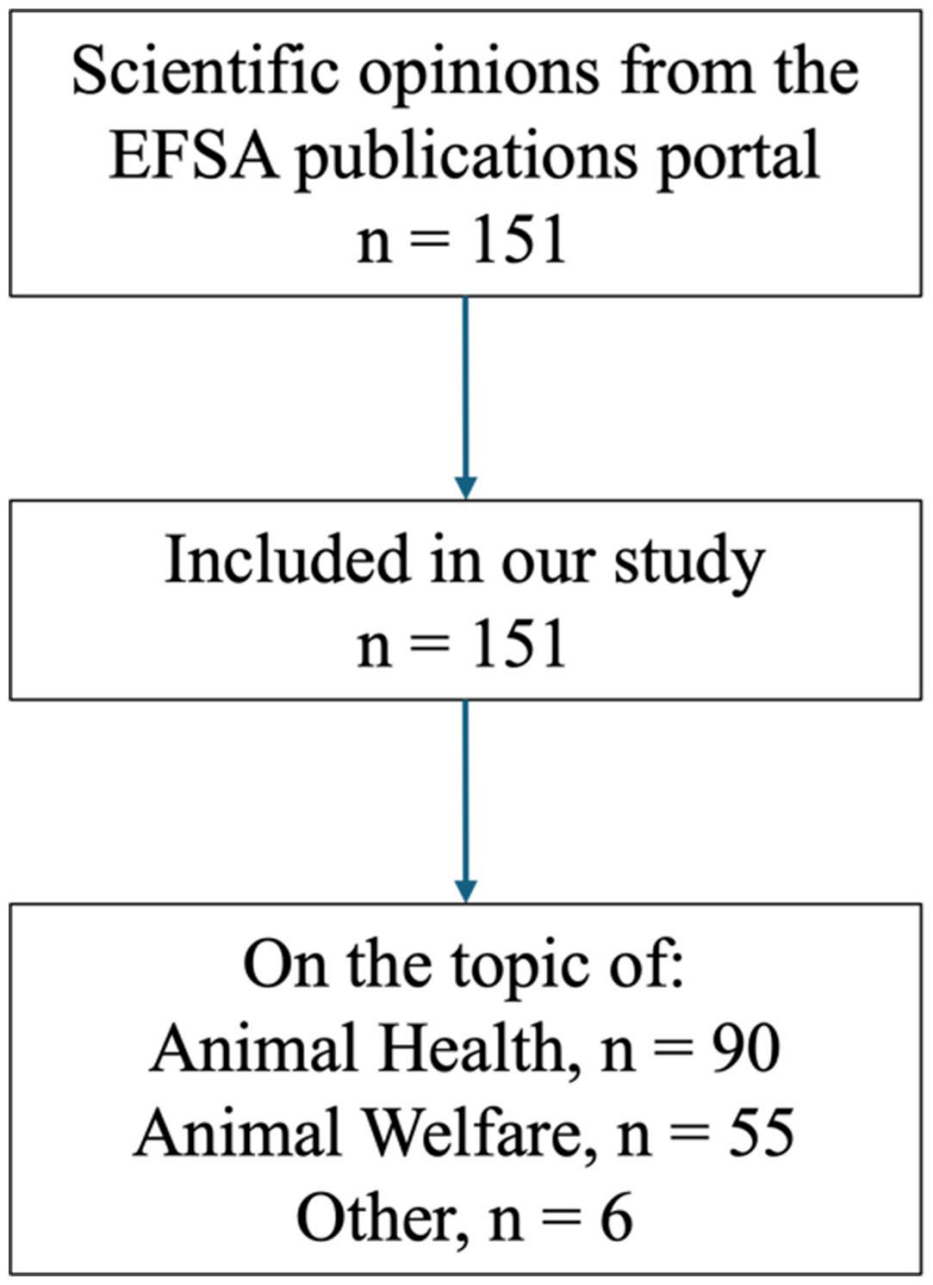
Study flow chart.

We included a total of 151 studies in our analysis (Figure 1). The earliest study was published in 2004, and nearly two thirds of the studies (98 out of 151, or 65%) have been published since 2017. Most studies (90, or 60%) focused on animal diseases, while 55 studies (36%) addressed animal welfare issues. The most studied species were pigs (*Sus scrofa domesticus*) and poultry (*Gallus gallus domesticus*), each featured in 25 studies (17%). Cattle (*Bos taurus*) were the focus of 23 studies, followed by horses (*Equus caballus*, 14 studies) and goats (*Capra hircus*, 12 studies). Rodents, such as rats (*Rattus norvegicus*) or mice (*Mus musculus*), were rarely studied, appearing in only 7 studies when considered within the broader category of mammals.

### Types of studies informing animal health and welfare policy

**Figure 2:**
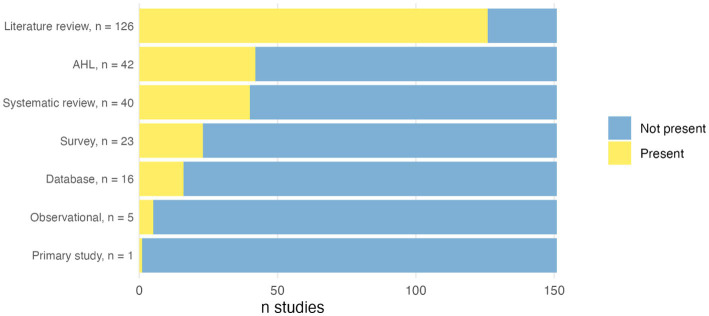
Overall proportion of study types among animal health and welfare EFSA reports. AHL, Animal Health Law.

Among the 151 studies reviewed, literature reviews were the most common type, present in 126 studies (83%) (Figure 2). Forty studies (27%) featured reviews which used at least one systematic review methodology according to our pre-specified criteria, therefore searching at least two databases, reporting a clear research question as well as inclusion and exclusion criteria, and the number of studies included in the data extraction process. Within this group, 11 studies (27%) were explicitly labelled as systematic reviews by their authors, while 25 (62%) were labelled by the authors as “extensive literature search.”

Other study types included surveys (23 studies, 15%), database studies (16 studies, 11%), and observational studies (5 studies, 3%) (more than one type of study could be included in a single report). Only one primary study was conducted. A total of 42 studies (28%) focused on assessing diseases following the AHL framework. If excluding these Animal Health Law studies, a study type not including systematic reviews, the proportion of systematic reviews was 37%.

**Figure 3:**
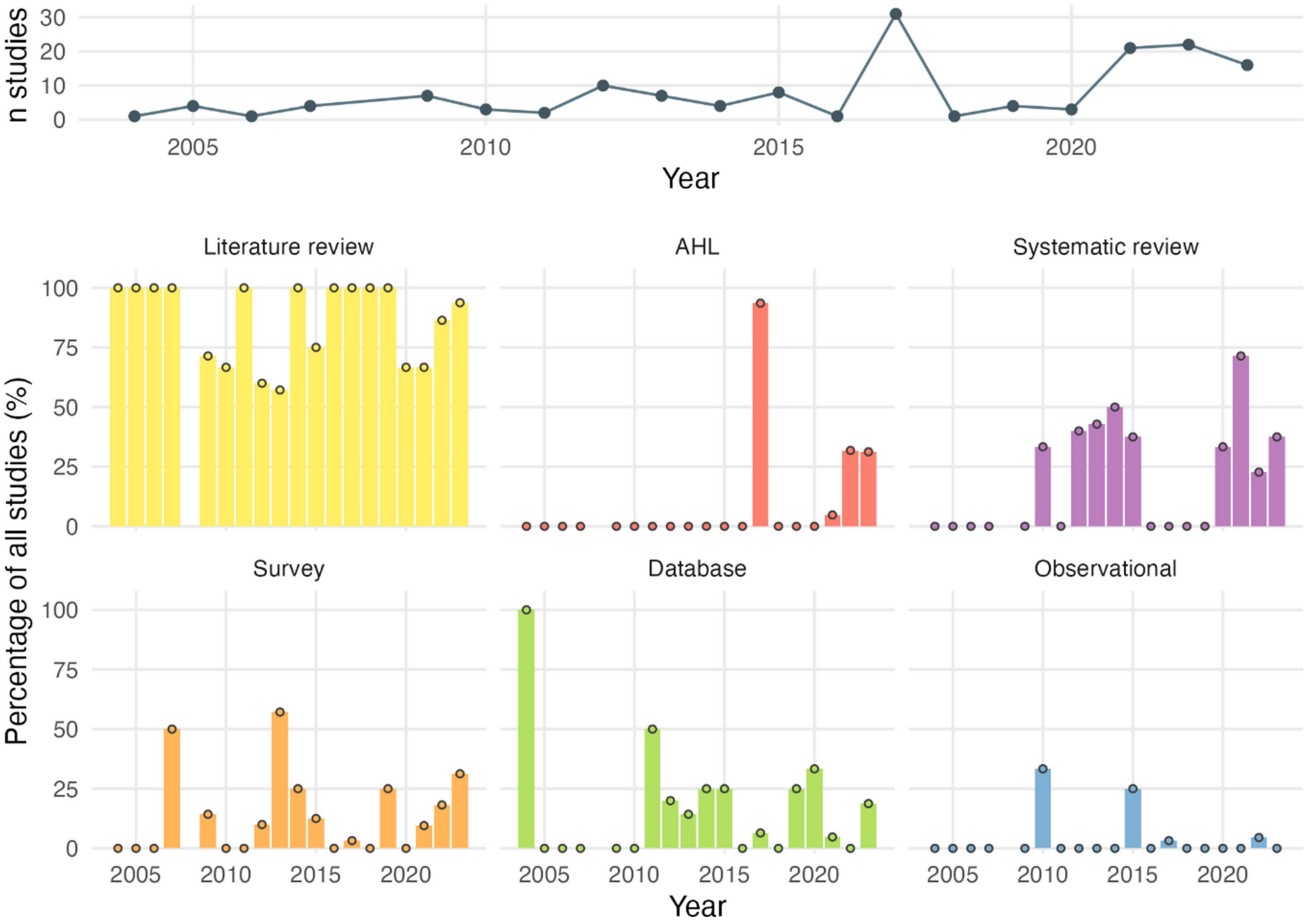
Proportion of study types over time. Percentages equal to 0 are shown as a dot. AHL, Animal Health Law.

Over time, the use of literature reviews has remained steady, while systematic reviews have gained popularity since 2010. In contrast, the use of other study types tended to decline (Figure 3).

### Reporting practices in reviews

**Figure 4:**
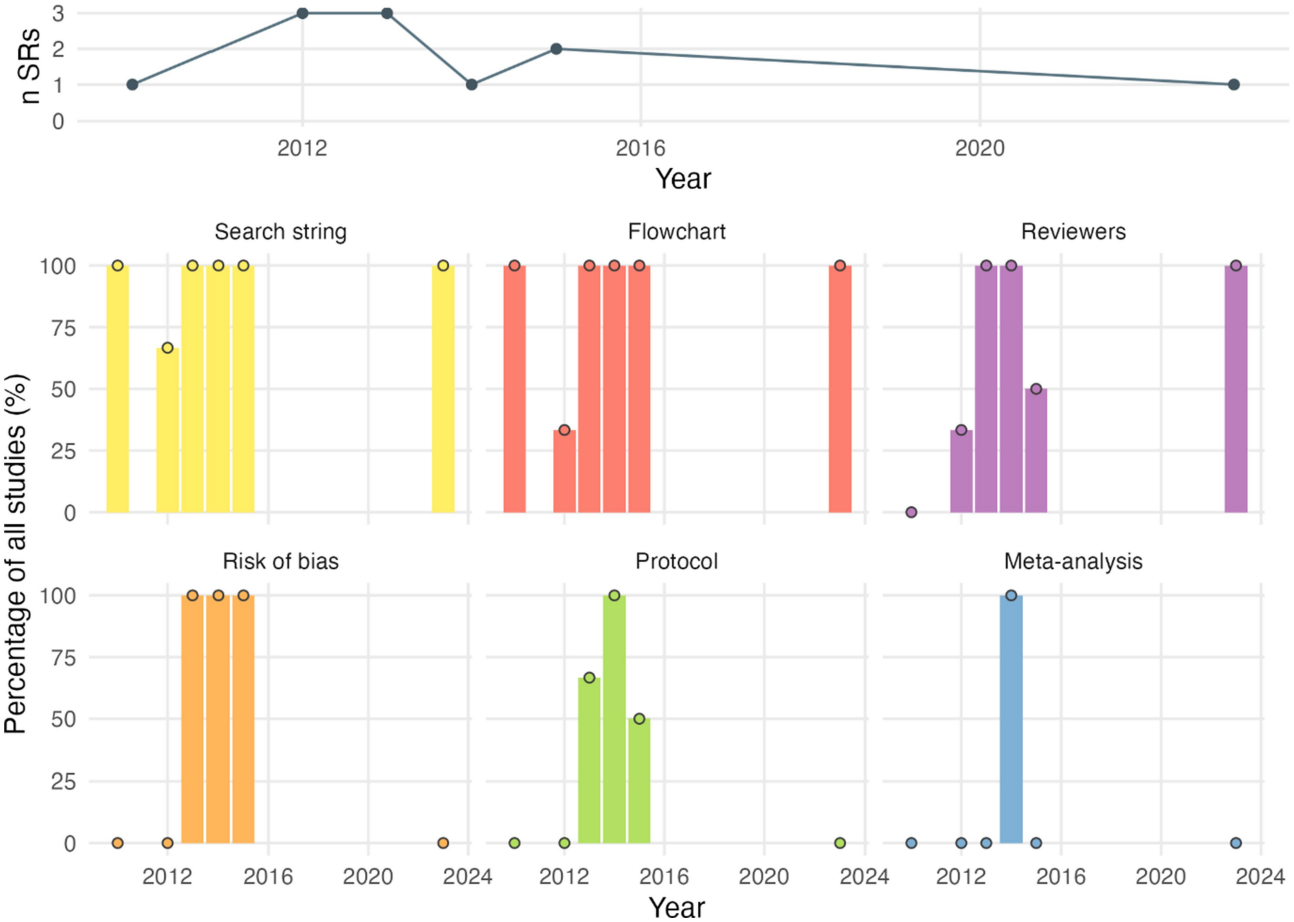
Reporting quality of systematic reviews. *Search string*: Authors explicitly reported a search string. *Flowchart*: Authors provided a study flow chart. *Reviewers*: At least abstract screening or data extraction was performed by at least 2 independent reviewers in duplicate. *Risk of bias*: A formal critical appraisal of included studies has been conducted. *Protocol*: An *a priori* study protocol has been drafted. *Meta-analysis*: A meta-analysis has been conducted complementing the systematic review. Percentages equal to 0 are shown as a dot. n SRs, number of systematic reviews per year.

If only considering the 11 systematic reviews as labelled by the authors, 10 provided a search string (91%), 9 a study flowchart (82%), 7 listed more than one reviewer for abstract screening and/or data extraction (64%), 5 reported a risk of bias assessment (45%), and 4 the a-priori registration of a study protocol (36%) (Figure 4). One study conducted a meta-analysis (labelled as such).

When stratified by topic, reporting quality items were assessed separately for systematic reviews addressing animal health and animal welfare (Supplementary Figures S1, S2). In both topic areas, similarly, only few studies reported protocol registration, risk of bias assessment, or meta-analysis, and no clear differences between topics were apparent.

## Discussion

### Main findings

The goal of this study was to assess the use and reporting quality of systematic review methodology in EFSA’s Scientific Opinions on animal health and welfare. Most EFSA reports focused on specific diseases, with slightly more than a third addressing animal welfare issues, primarily in farm animals like pigs, poultry, and cattle. Among the most frequent types of studies informing these reports were different types of literature reviews including systematic reviews. The reporting quality of systematic reviews was moderate, with only few reporting details on database search and an a-priori registration of a study protocol.

### Findings in the context of existing evidence

Starting from 2021, there was a notable increase in EFSA scientific opinions. This rise can be attributed to several factors. First, the implementation of the Transparency Regulation in 2021 mandated more comprehensive documentation and publication of scientific assessments, thereby increasing the number of reports.^3^ Second, EFSA’s strategic objectives for 2027 prioritize food safety as part of sustainable food systems, requiring thorough assessments of new risks and technologies, and fostering greater collaboration with stakeholders. Furthermore, regulatory and societal changes aimed at creating a fair, healthy, and environmentally-friendly food system have required detailed monitoring and reporting, contributing to the rise in reports. Third, advancements in technology and data collection methods have enabled EFSA to gather and analyze more data, supporting the production of more comprehensive food safety reports. Lastly, the COVID-19 pandemic underscored the importance of scientific research and risk analysis in public health (20), driving up demand for such food safety reports and leading to increased scrutiny of food safety practices.

The increase in EFSA reports coincided with the growing adoption of systematic review methodology, even in more traditional, non-systematic literature reviews. Elements such as the inclusion of detailed search strategies, searching multiple databases for relevant literature, critically appraising the included studies, and registering study protocols became more common, with about a quarter to a third of all reports incorporating these methods. Known for their rigor, systematic reviews provide reliable, less biased findings by synthesizing results from multiple studies (21, 22). With this, systematic reviews and meta-analyses are considered the highest level of evidence (1). They act as a lens through which other studies are appraised and synthesized (23), helping policymakers understand what is known and unknown about a certain topic. This comprehensive understanding aids in making informed, evidence-based decisions and reduces the risk of policy failure due to unforeseen consequences or unaddressed issues (24). Furthermore, systematic reviews can highlight areas where evidence is lacking, guiding future research priorities (8). Policymakers can use this information to support necessary studies. Additionally, systematic reviews can be updated with new findings, ensuring that policies remain relevant over time.

The systematic reviews identified in the reports had moderate reporting quality: some studies failed to report using dedicated search queries or identified the actual literature databases searched. This aligns with recent analyses showing a high prevalence of less rigorous preclinical systematic reviews (12, 25–28). A well-documented and comprehensive search, including the full search query with syntax and a clear specification of the literature databases used, is essential for enabling update searches and ensuring reproducibility (4). The limited use of risk of bias assessment means that policymakers may not have the tools to judge the reliability of individual studies, which can affect the weighting of evidence and the robustness of resulting animal health and welfare measures. Notably, only about one-third of systematic reviews reported a pre-registered study protocol. This may partly reflect current publication practices at EFSA, where protocols may exist but are not routinely made public. Nevertheless, the limited availability of protocols restricts the transparency and reproducibility of the review process and constrains external assessment of methodological decisions.

What is the potential impact of lacking these key items in systematic reviews? Relatively poor reporting, such as missing search queries, unclear database specifications, or failure to pre-register a protocol, has been shown to be associated with a lack of actually performing these measures (29). This gap in transparency undermines the reliability of conclusions and increases the risk of bias. As a result, policy decisions based on such systematic reviews may be influenced by incomplete or flawed evidence (14, 30, 31).

### Recommendations

We propose the following two recommendations: First, dedicated educational efforts should be implemented to enhance the rigor of systematic reviews among EFSA and other stakeholders involved in animal welfare policy, including support for EFSA’s ongoing initiatives to improve review quality (32, 33). Second, automating key steps in the systematic review process, such as data extraction and study screening, could help manage the growing volume of evidence while ensuring timely and well-informed assessments (4), using existing dedicated software solutions such as Rayyan (34) or Covidence (35).

### Limitations

Findings should be interpreted considering several limitations. First, we focused on scientific opinions, which might result in the over-representation of systematic reviews. Additionally, we explicitly looked for the term “systematic review” but also included studies that met four specific criteria when the term was not identified, namely if a specific research question, inclusion and exclusion criteria for studies, at least two databases were searched, and the total number of studies were reported. This approach might have skewed our analysis. Second, scientific opinions are not policy documents themselves but provide independent scientific advice for policymakers (14, 36). Nevertheless, while this study indirectly measures their impact on policy, these opinions often serve as a foundation for policy decisions. Third, we did not classify individual reports based on their purpose, which could introduce bias since certain study purposes might not benefit from a systematic review and instead require other dedicated methodologies, such as database studies. This might lead to an underestimation of the contribution of systematic reviews to animal welfare policy. Fourth, we assessed the reporting rigor of the systematic reviews rather than their actual rigor. It is possible that most systematic reviews used dedicated search queries, but only a minority reported them in the final report. Finally, our analysis was restricted to scientific opinions issued by the EFSA. EFSA is the central EU authority for risk assessment and its scientific opinions inform EU-wide food and feed legislation as well as national measures. As such, these outputs provide a relevant case study but may not fully reflect how systematic review methods are used in other regulatory settings. Nonetheless, our approach is not specific to EFSA and could be transferred to scientific outputs from other authorities (e.g., the US Food and Drug Administration, Health Canada/Canadian Food Inspection Agency, the UK Food Standards Agency, ANSES, BfR) to evaluate the generalizability of our findings and enable cross-agency comparisons in future work.

### Strengths

This study provides complete coverage of EFSA scientific opinions, summarizing current practices and trends in evidence synthesis within these documents. Additionally, it includes a detailed assessment of key reporting items in systematic reviews.

## Conclusion

Systematic review methodology is increasingly used in EFSA’s Scientific Opinions on animal health and welfare. However, reporting and methodological rigor remain inconsistent. Strengthening standards for transparency and protocol registration would improve reproducibility and support more consistent, evidence-based policy advice.

## Data Availability

The datasets presented in this study can be found in online repositories. The names of the repository/repositories and accession number(s) can be found at: https://osf.io/t5csg/.

## Footnotes

1
https://osf.io/t5csg/

2
https://www.efsa.europa.eu/en/publications

3
https://op.europa.eu/webpub/efsa/strategy-2027/en/

## References

[1] Higgins JP Thomas J Chandler J Cumpston M Li T Page MJ . Cochrane handbook for systematic reviews of interventions. Hoboken, NJ: John Wiley & Sons (2019). 10.1002/14651858.ED000142 PMC10284251 31643080

[2] de Vries RB Wever KE Avey MT Stephens ML Sena ES Leenaars M. The usefulness of systematic reviews of animal experiments for the design of preclinical and clinical studies. ILAR J. (2014) 55:427–37. doi: 10.1093/ilar/ilu043, 25541545 PMC4276599

[3] Rosso M Doneva SE Howells DW Leenaars CH Ineichen BV. Summer school for systematic reviews of animal studies: fostering evidence-based and rigorous animal research. ALTEX. (2024) 41:131–4. doi: 10.14573/altex.2310251, 38204181

[4] Ineichen BV Held U Salanti G Macleod MR Wever KE. Systematic review and meta-analysis of preclinical studies. Nat Rev Methods Primers. (2024) 4:72. doi: 10.1038/s43586-024-00347-x

[5] Bugajska JV Hild BF Brüschweiler D Meier ED Bannach-Brown A Wever KE . How long does it take to complete and publish a systematic review of animal studies? BMC Med Res Methodol. (2025) 25:226. doi: 10.1186/s12874-025-02672-5, 41034728 PMC12487012

[6] Ritskes-Hoitinga M Pound P. The role of systematic reviews in identifying the limitations of preclinical animal research, 2000–2022: part 1. J R Soc Med. (2022) 115:186–92. doi: 10.1177/01410768221093551, 35502678 PMC9069614

[7] Ritskes-Hoitinga M Pound P. The role of systematic reviews in identifying the limitations of preclinical animal research, 2000–2022: part 2. J R Soc Med. (2022) 115:231–5. doi: 10.1177/01410768221100970, 35616311 PMC9158443

[8] Ineichen BV Furrer E Grüninger SL Zürrer WE Macleod MR. Analysis of animal-to-human translation shows that only 5% of animal-tested therapeutic interventions obtain regulatory approval for human applications. PLoS Biol. (2024) 22:e3002667. doi: 10.1371/journal.pbio.3002667, 38870090 PMC11175415

[9] Perel P Roberts I Sena E Wheble P Briscoe C Sandercock P . Comparison of treatment effects between animal experiments and clinical trials: systematic review. BMJ. (2007) 334:197. doi: 10.1136/bmj.39048.407928.BE, 17175568 PMC1781970

[10] Besselink MG van Santvoort HC Buskens E Boermeester MA van Goor H Timmerman HM . Probiotic prophylaxis in predicted severe acute pancreatitis: a randomised, double-blind, placebo-controlled trial. Lancet. (2008) 371:651–9. doi: 10.1016/S0140-6736(08)60207-X, 18279948

[11] Hooijmans CR de Vries RB Rovers MM Gooszen HG Ritskes-Hoitinga M. The effects of probiotic supplementation on experimental acute pancreatitis: a systematic review and meta-analysis. PLoS One. (2012) 7:e48811. doi: 10.1371/journal.pone.0048811 23152810 PMC3496732

[12] van Luijk J Bakker B Rovers MM Ritskes-Hoitinga M de Vries RB Leenaars M. Systematic reviews of animal studies; missing link in translational research? PLoS One. (2014) 9:e89981. doi: 10.1371/journal.pone.0089981 24670965 PMC3966727

[13] Russell WMS Burch RL. The principles of humane experimental technique. London: Methuen (1959).

[14] Berthe F Vannier P Have P Serratosa J Bastino E Maurice Broom D . The role of EFSA in assessing and promoting animal health and welfare. EFSA J. (2012) 10:s1002. doi: 10.2903/j.efsa.2012.s1002

[15] European Food Safety Authority. (2024) EFSA homepage.

[16] E-Parliament. (2002). Regulation (EC) no 178/2002 of the European Parliament and of the council of 28 January 2002 laying down the general principles and requirements of food law, establishing the European food safety authority and laying down procedures in matters of food safety. pp. L31–L24.

[17] Liesner J. Rosso M. Ineichen B. (2024) How commonly do systematic reviews inform EFSA policy? Available online at: https://osf.io/t5csg/ (Accessed November 24, 2025).

[18] R.C. Team. A language and environment for statistical computing. Vienna: R Foundation for Statistical Computing (2023).

[19] EFSA Panel on Animal Health and Welfare (AHAW) Welfare S More A Bøtner A Butterworth A Calistri P . Ad hoc method for the assessment on listing and categorisation of animal diseases within the framework of the animal health law. EFSA J. (2017) 15:e04783. doi: 10.2903/j.efsa.2017.4783, 32625537 PMC7010140

[20] Raynaud M Goutaudier V Louis K Al-Awadhi S Dubourg Q Truchot A . Impact of the COVID-19 pandemic on publication dynamics and non-COVID-19 research production. BMC Med Res Methodol. (2021) 21:1–10. doi: 10.1186/s12874-020-01190-w, 34809561 PMC8607966

[21] Sena ES Currie GL McCann SK Macleod MR Howells DW. Systematic reviews and meta-analysis of preclinical studies: why perform them and how to appraise them critically. J Cereb Blood Flow Metab. (2014) 34:737–42. doi: 10.1038/jcbfm.2014.28, 24549183 PMC4013765

[22] Soliman N Rice AS Vollert J. A practical guide to preclinical systematic review and meta-analysis. Pain. (2020) 161:1949–54. doi: 10.1097/j.pain.0000000000001974, 33449500 PMC7431149

[23] Murad MH Asi N Alsawas M Alahdab F. New evidence pyramid. BMJ. Evid Based Med. (2016) 21:125–7. doi: 10.1136/ebmed-2016-110401, 27339128 PMC4975798

[24] Gough D. Oliver S. Thomas J., Learning from research: Systematic reviews for informing policy decisions. (2013).

[25] Hooijmans CR Donders R Magnuson K Wever KE Ergün M Rooney AA . Assessment of key characteristics, methodology, and effect size measures used in meta-analysis of human-health-related animal studies. Res Synth Methods. (2022) 13:790–806. doi: 10.1002/jrsm.1578, 35679294 PMC9796290

[26] Langendam MW Magnuson K Williams AR Walker VR Howdeshell KL Rooney AA . Developing a database of systematic reviews of animal studies. Regul Toxicol Pharmacol. (2021) 123:104940. doi: 10.1016/j.yrtph.2021.104940, 33964349 PMC11364211

[27] Hunniford VT Montroy J Fergusson DA Avey MT Wever KE McCann SK . Epidemiology and reporting characteristics of preclinical systematic reviews. PLoS Biol. (2021) 19:e3001177. doi: 10.1371/journal.pbio.3001177, 33951050 PMC8128274

[28] Hild BF Brüschweiler D Hild STK Bugajska J von Wyl V Rosso M . Quality, topics, and demographic trends of animal systematic reviews-an umbrella review. J Transl Med. (2025) 23:21. doi: 10.1186/s12967-024-05992-0, 39762882 PMC11702210

[29] Boutron I Guittet L Estellat C Moher D Hróbjartsson A Ravaud P. Reporting methods of blinding in randomized trials assessing nonpharmacological treatments. PLoS Med. (2007) 4:e61. doi: 10.1371/journal.pmed.0040061, 17311468 PMC1800311

[30] Budolfson M Fischer B Scovronick N. Animal welfare: methods to improve policy and practice. Science. (2023) 381:32–4. doi: 10.1126/science.adi0121, 37410843

[31] EFSA Panel on Animal Health and Welfare Nielsen SS Alvarez J Bicout DJ Calistri P Canali E . Methodological guidance for the development of animal welfare mandates in the context of the farm to fork strategy. EFSA J. (2022) 20:e07403. doi: 10.2903/j.efsa.2022.7403, 35846109 PMC9275173

[32] European Food Safety Authority. Application of systematic review methodology to food and feed safety assessments to support decision making. EFSA J. (2010) 8:1637. doi: 10.2903/j.efsa.2010.1637 PMC13085193 42006960

[33] European Food Safety Authority. Tools for critically appraising different study designs, systematic review and literature searches. EFSA Support Publ. (2015) 12:836E. doi: 10.2903/sp.efsa.2015.EN-836

[34] Ouzzani M Hammady H Fedorowicz Z Elmagarmid A. Rayyan—a web and mobile app for systematic reviews. Syst Rev. (2016) 5:210. doi: 10.1186/s13643-016-0384-4, 27919275 PMC5139140

[35] Kellermeyer L Harnke B Knight S. Covidence and rayyan. J Med Lib Assoc. (2018) 106:580. doi: 10.5195/jmla.2018.513, 39022435

[36] Ribó O Candiani D Serratosa J. Role of the European food safety authority (EFSA) in providing scientific advice on the welfare of food producing animals. Ital J Anim Sci. (2009) 8:9–17. doi: 10.4081/ijas.2009.s1.9

