# Supplementary material for: Use and reporting of systematic review methodology in EFSA scientific opinions on animal health and welfare
Source: Front Vet Sci. 2026 Jan 19;12:1594235. doi: 10.3389/fvets.2025.1594235 (PMC12862926; doi:10.3389/fvets.2025.1594235)

**Use and reporting of systematic review methodology in EFSA Scientific Opinions on animal health and welfare**

Johann Liesner^1,2^, Benjamin V. Ineichen^1,2^*, Marianna Rosso^1^*

**Short title**: Animal welfare policy are commonly informed by systematic review

**Author affiliations:**

^1^ Center for Reproducible Science and Research Synthesis, University of Zurich, Zurich, Switzerland

^2^ Department of Clinical Research, University of Bern, Bern, Switzerland

*Authors share senior authorship

**Correspondence to**:

Benjamin Victor Ineichen, University of Bern, Department of Clinical Research, Bern, Switzerland, ORCID: 0000-0003-1362-4819

[benjamin.ineichen@unibe.ch](mailto:benjamin.ineichen@unibe.ch)

**Supplementary data**

**Figure S1**: Reporting quality of systematic reviews on the topic of animal health.


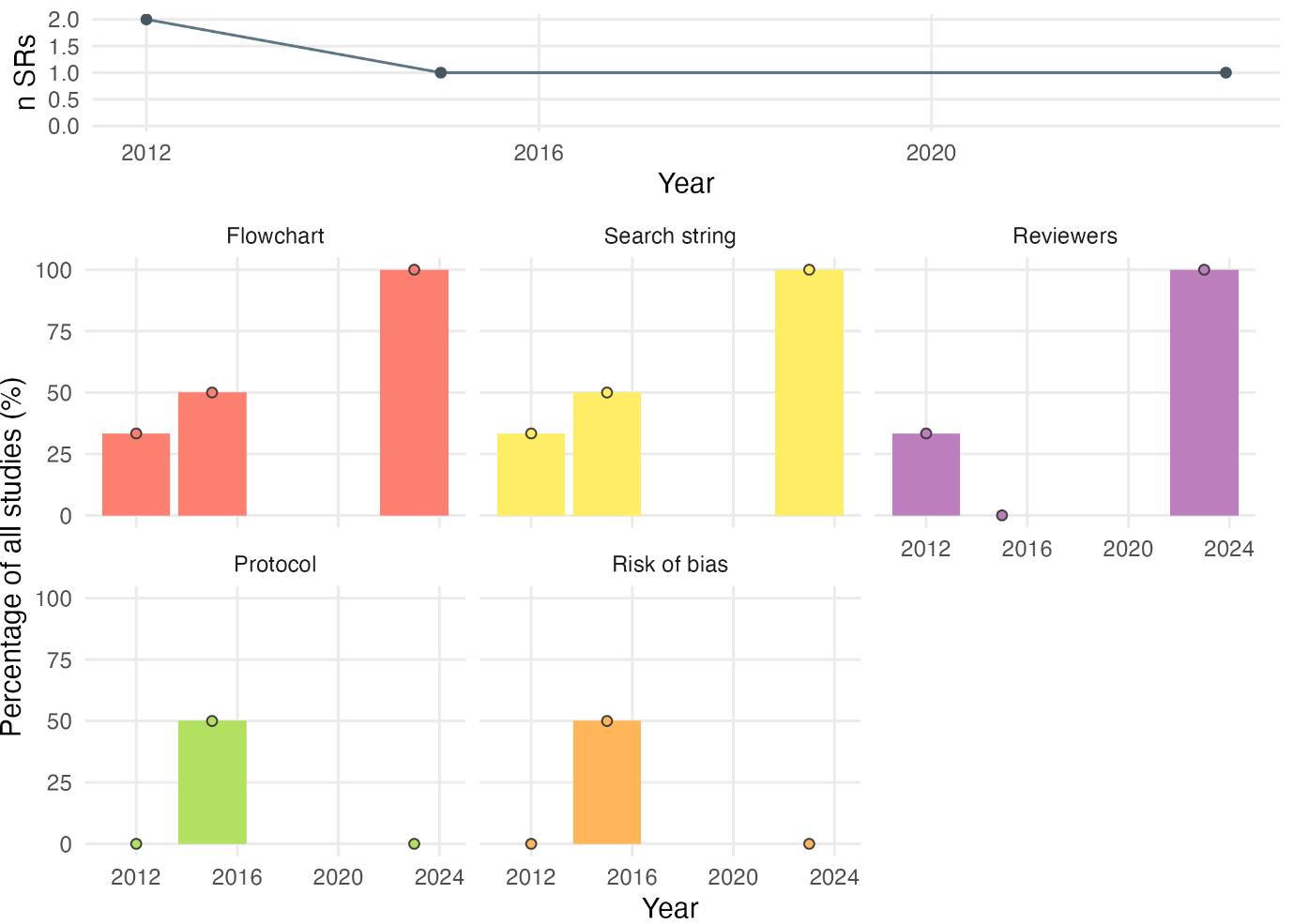


**Figure S2**: Reporting quality of systematic reviews on the topic of animal welfare.


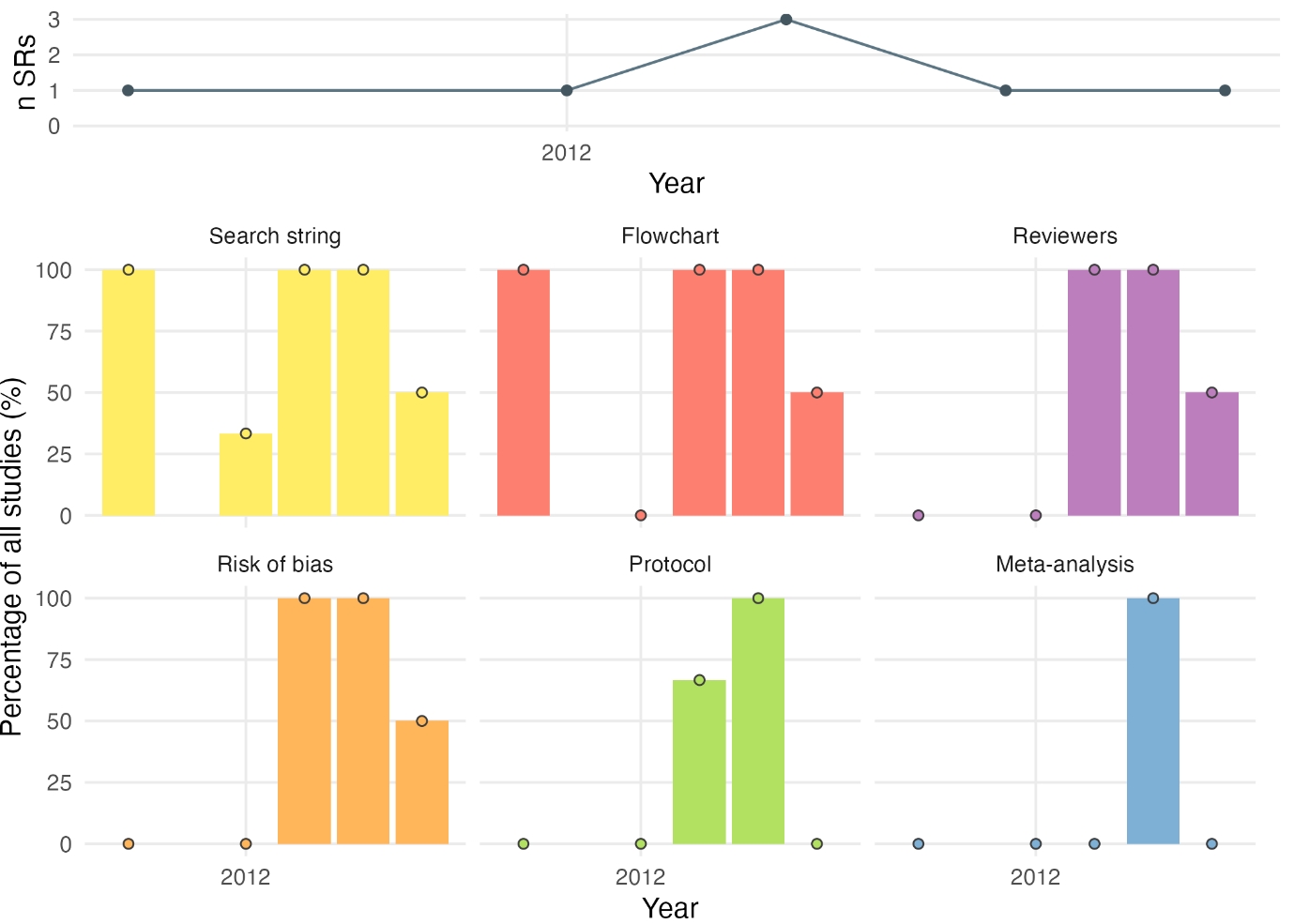

Supplement: Supplementary file 1 [file Data_Sheet_1.docx]
